# Research on the correlation between the size of condyle and occlusion plane in skeletal Class II malocclusions

**DOI:** 10.1002/cre2.672

**Published:** 2022-10-13

**Authors:** Hainan Hu, Wanrong Lin, Jun Wu, Haisong Xu

**Affiliations:** ^1^ Department of Orthodontics, Qujing First People's Hospital Qujing Hospital affiliated to Kunming Medical University Yunnan China; ^2^ Department of Orthodontics, Shanghai Ninth Peoples's Hospital Shanghai Jiao Tong University, School of Medicine Shanghai China; ^3^ Shanghai Key Laboratory of Stomatology, National Center for Stomatology, National Clinical Research Center for Oral Diseases Shanghai Jiao Tong University Shanghai China; ^4^ Department of Public Health in Epidemiology New York University New York New York USA; ^5^ Department of Plastic and Reconstructive Surgery, Shanghai Ninth People's Hospital Shanghai Jiao Tong University School of Medicine Shanghai People's Republic of China

**Keywords:** anteroposterior diameters of the condyle, keletal Class II, medial‐lateral diameter, occlusal plane angle

## Abstract

**Objectives:**

This study was designed to investigate the relationship between the morphological structure of condyle and occlusal plane in skeletal Class II malocclusions by imaging measurement.

**Materials and Methods:**

This study included 65 skeletal Class II adult patients (18–35 years old) who met the criteria, and all were taken with cone beam computed tomography (CBCT) images (skeletal Class II high angle 38 cases, average angle 18 cases, and low angle nine cases). The statistical methods of mean standard deviation, Pearson correlation, and analysis of variance were used to study the correlation between the size of the condyle and occlusal plane in skeletal Class II malocclusion.

**Results:**

The FMA and SN‐OP between the groups in skeletal Class II malocclusion are considered statistically significant, *p* < .05 high angle group > average angle group > low angle group, whereas there are significant correlations between FMA, FH‐OP, SN‐OP, and the medial–lateral diameter (MLD) of the condyle, *p* < .05, showing a negative correlation. The anteroposterior diameter of the condyle has no significant correlation with these angles, and the high‐angle group size is smaller than the other groups.

**Conclusion:**

In patients with skeletal Class II high angle malocclusion, the MLD and anteroposterior diameters of condyle were smaller than those of average angle and low angle groups, and negatively correlated with the FMA and SN‐OP. That is the steeper occlusal plane, the smaller MLD of the condyle. It suggests whether orthodontists can promote the stability of the morphological structure of the condyle by changing the inclination of the occlusal plane during the orthodontic process.

## 1 INTRODUCTION AND BACKGROUND

Skeletal Class II malocclusion is a common type of malocclusion in the clinic, which is characterized by the sagittal upward relationship of the maxilla and mandible, accompanied by vertical malocclusion. However, the relationship between malocclusion and the temporomandibular joint (TMJ) is still in conflict. Abnormal occlusion may cause temporomandibular disorders (TMD) has been basically recognized, and the influence of occlusal relationship on the morphology of TMJ is still controversial. Some studies try to reveal the correlation between occlusal factors and mandibular joint morphology, but others believe that there is no correlation between them. However, the changes in the internal morphology and structure of the TMJ can affect the growth and development of the mandible by affecting the condyle. The condyle is an important growth and reconstruction center of the mandible, and it is a key part of transmitting bite force. When an abnormal occlusal relationship exists, the condyle will undergo adaptive reconstruction after being stimulated by stress, showing different morphological structures and sizes, which is very likely to affect the morphological development of the TMJ, and then cause abnormal mandibular development (AI Taki et al., 2015). In recent years, many scholars have conducted research on the location of the condyle and found that facial growth, occlusal changes, and maxillofacial functional or pathological changes can affect the location of the condyle (Paknahad & Shahidi, 2017; Paknahad et al., 2016; Weiping & Haiyuan, 2015; Yin et al., 2017). A large number of articles have also studied the position and structure of the TMJ in different occlusal relationships and skeletal relationships (Ueki et al., 2008; Vitral et al., 2004). Scholars have explored the causes of this type of malocclusion from different angles. However, most scholars have focused on the study of the morphological characteristics of the submandibular joints in patients with different skeletal malocclusions, and there are few studies on the influence of the steep slope of the occlusal plane on the size of the condyle (Di Venere et al., 2016; Osborn, 1987).

In this study, adult patients with skeletal Class II malocclusion were selected as the research object, and the high angle, average angle, and low angle skeletal Class II malocclusion were grouped as the research object to explore whether the condyle size of adult patients with skeletal Class II malocclusion was related to the occlusal plane. To provide definite guidance for orthodontists in clinical examination, scheme design, and prognosis judgment.

## 2 PATIENTS AND METHODS

### 2.1 Research object

A total of 65 healthy adult patients with normal permanent teeth and skeletal Class II were selected in our department. Cone beam computed tomography (CBCT) was taken after the patient and his family knew and agreed, and the ANB, FMA, SN‐OP, and FH‐OP angles were measured to group the study subjects. Meet the following inclusion criteria: 65 cases of young skeletal Class II malocclusion between 18 and 35 years old, 29 males and 36 females; The sagittal bone surface type was skeletal Class II (ANB ≥ 4.7°); the vertical bone surface type was the high angle in 38 cases (FH‐MP ≥ 32°), and the average angle was 18 cases (22° < FH‐MP < 32°), nine cases of low angle (FH‐MP ≤ 22°); Normal permanent dentition, excluding the history of orthognathic surgery, cleft lip, and parotid, history of submandibular joint disease, maxillofacial trauma, tumor, and other diseases that can cause maxillofacial asymmetry deformity; The whole body is in good health.

The authors are accountable for all aspects of the work in ensuring that questions related to the accuracy or integrity of any part of the study are appropriately investigated and resolved. All procedures performed in the study involving human participants were as per the ethical standards of the institutional and/or national research committee (s) and the Helsinki Declaration (as revised in 2013). This study was approved by the Ethics Review Committee of our hospital. Radiographs and participation in the study were authorized by the patient's parents. Informed consent was obtained from the participant with a detailed description of the purpose and benefits of the study.

### 2.2 Software and equipment

NewTom VG‐AFP CBCT (Radiation posthumous 50 mSv, scanning thickness 0.15 mm). In this study, CBCT was used to measure and analyze the anteroposterior diameters and MLD of the condyle in adult patients with skeletal Class II malocclusion.

### 2.3 Experimental method

**Figure 1:**
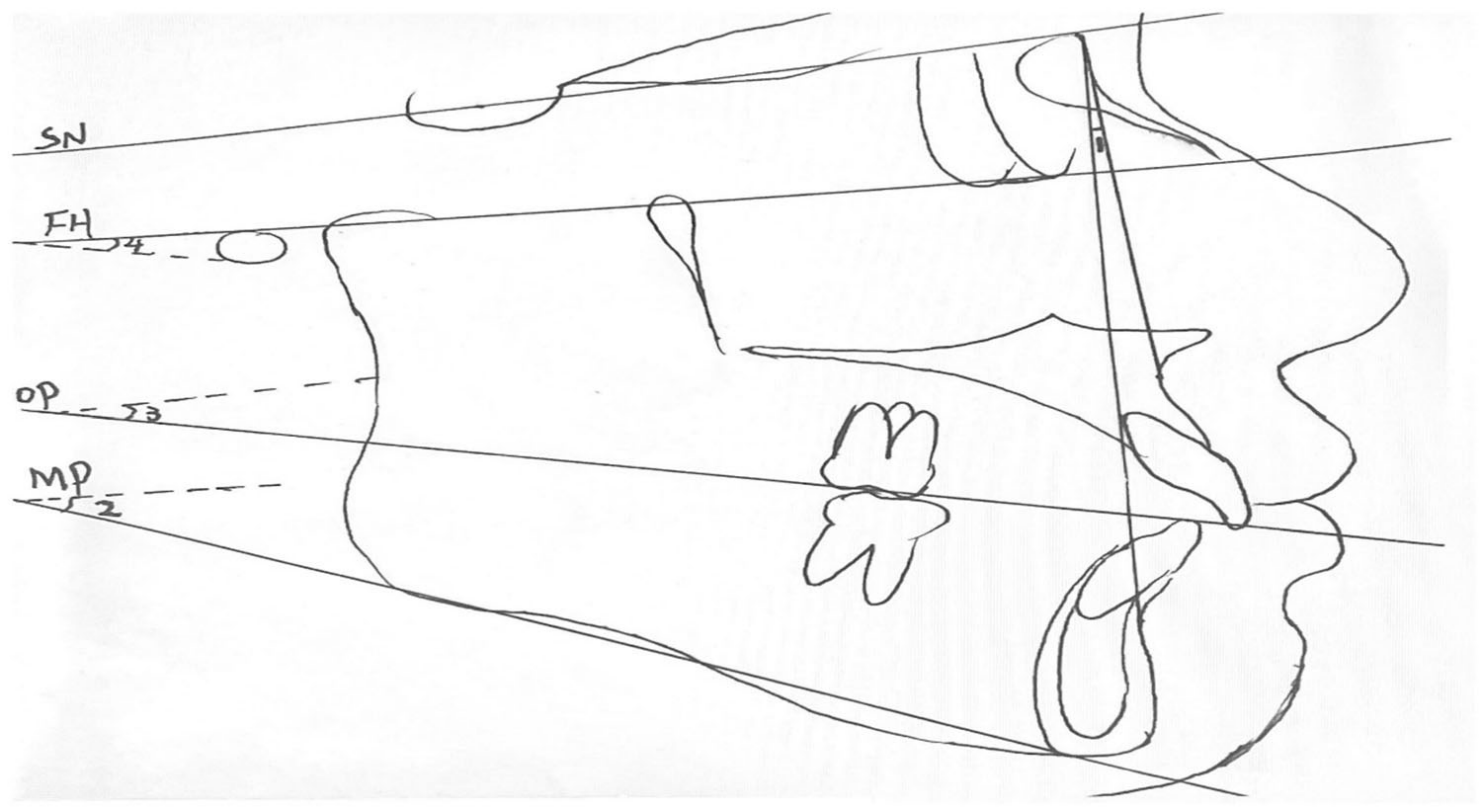
Cephalometric Indexes. 1, ANB angle; 2, FH‐MP angle; 3, FH‐OP angle; 4, SN‐OP angle.

**Figure 2:**
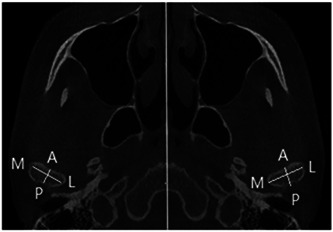
Measurement indexes of condyle. AP, anteroposterior diameter of the condyle; ML, MLD of condyle.

All research subjects were scanned using CBCT in the Department of Radiology of our hospital, and the scanning conditions were: Voltage 85 kV, current 21 mA, and tomographic thickness 0.15 mm. Select and group the research objects according to the size of ANB angle and FH‐MP, and then measure the FH‐OP angle and SN‐OP angle (occlusal plane angle) of each group respectively (Figure 1). The axial plane of the condyle is determined by referring to the maximum cross‐sectional area of the condyle, and the condyle is sliced. The center point of the layer tangent coincides with the center point of the condyle, and the layer tangent is perpendicular to the long axis of the condyle, symmetrical left and right, and the slicing thickness is 0.3 mm. Measurement of the axial surface of the condyle: the length of the long axis of the condyle: the maximum inner and outer diameter of the condyle; the length of the short axis of the condyle: the largest anteroposterior diameter of the condyle, which is used to analyze the relevant parameters of the largest mesial and distal diameter and the maximum inner and outer diameter of the condyle (Figure 2).

## 3 RESULTS

**Figure 3:**
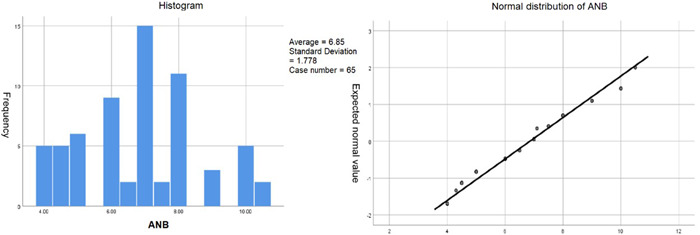
Histogram and Q–Q diagram

Due to the small sample size, it can be seen that it is a roughly normal distribution according to the graphic method (histogram and Q–Q diagram) (Figure 3). Excel 2010 is used to complete the data entry and sorting. In the next step, the mean standard deviation is used for description, Pearson correlation and analysis of variance, and other parameter tests.

As can be seen from Table 1, a grouping of research objects and measurement items.

**Table 1:** Grouping of research objects and measurement items

Measurement items	Definition
ANB angle	ANB ≥ 4.7° (skeletal Class II)
FMA angle	Mandibular plane angle (high angle FMA ≥ 32°; average angle 22° < FMA < 32°; Low angle FMA ≤ 22°)
0P plane	Evenly divide the posterior occlusal contact points
FH‐OP	The angle of intersection between frank fort plane and occlusal plane
SN‐OP (occlusal plane angle)	The angle of intersection between skull base plane and occlusal plane
Maximum MLD of the condyle	Length of the long axis of the condyle
Maximum anteroposterior diameter of the condyle	Length of the short axis of the condyle

As can be seen from Table 2, difference analysis in FMA and SN‐OP and different angle groups were significant, *p* < .05, their sizes were high angle group > average angle group > low angle group; Difference analysis in MLD of the condyle and different angle groups were significant, *p* < .05. From the mean value of different angle groups, it can be seen that the MLD of the condyle in high angle group < average angle group < low angle group. The MLD and anteroposterior diameters of the condyle in the high angle group were the smallest; There were no significant analysis differences in the anteroposterior diameter of the condyle and different angle groups (MLD of the normal condyle is about 15–20 mm, and the anteroposterior diameter is about 10 mm). The measurement results of the MLD of the condyle in the experimental group were within the normal value, while the measurement results of the anteroposterior diameter of the condyle were smaller than the normal value.

**Table 2:** Overall correlation analysis

Measurement items	High angle data mean (SD)	Low angle data mean (SD)	Average angle data mean (SD)	*p*
ANB	7.02 (1.81)	5.72 (1.23)	7.07 (1.80)	0.120
FMA	35.99 (4.55)	19.22 (2.33)	26.84 (1.89)	<0.001
FH‐OP	12.37 (5.91)	7.00 (3.77)	10.36 (7.48)	0.061
SN‐OP	20.61 (6.04)	16.11 (4.91)	17.28 (5.68)	0.041
MLD of the condyle (left side)	16.78 (2.41)	19.36 (1.90)	18.21 (2.05)	0.005
MLD of the condyle (right side)	16.74 (2.86)	19.32 (1.95)	17.00 (2.15)	0.030
Anteroposterior diameter of the condyle (left side)	8.10 (1.57)	8.21 (1.89)	8.16 (1.37)	0.978
Anteroposterior diameter of the condyle (right side)	7.70 (1.66)	8.39 (1.36)	8.67 (1.20)	0.070

As can be seen from Table 3, there was a significant correlation between FMA, FH‐OP, SN‐OP, and the MLD of the condyle. The anteroposterior diameter of the condyle was not significantly correlated with FMA, FH‐OP, and SN‐OP.

**Table 3:** Correlation analysis among high angle group, average angle group, and low angle group

Correlation	ANB		FMA		FH‐OP		SN‐OP	
	*r*	*p*	*r*	*p*	*r*	*p*	*r*	*p*
MLD of the condyle (left)	−0.365	0.003	−0.511	<0.001	−0.448	<0.001	−0.432	<0.001
MLD of the condyle (right)	−0.244	0.050	−0.302	0.015	−0.438	<0.001	−0.228	0.068
MLD of the condyle (mean)	−0.327	0.008	−0.435	<0.001	−0.048	<0.001	−0.352	0.004
MLD of the condyle (left)	0.109	0.398	−0.052	0.681	0.025	0.841	−0.005	0.966
MLD of the condyle (right)	0.010	0.940	−0.224	0.073	0.006	0.960	−0.076	0.547
MLD of the condyle (mean)	0.068	0.593	−0.158	0.208	0.018	0.886	−0.047	0.711

As can be seen from Table 4, in the high angle group, FMA, SN‐OP, and FH‐OP were significantly correlated with the MLD of the condyle (left side), which was negatively correlated; FH‐OP was negatively correlated with the anteroposterior diameter (right side) of the condyle; FMA, SN‐OP, and FH‐OP had no significant correlation with the MLD of the condyle (right) and the anteroposterior diameter of the condyle (left).

**Table 4:** Intragroup correlation analysis of high angle

Correlation	ANB		FMA		FH‐OP		SN‐OP	
	*r*	*p*	*r*	*p*	*r*	*p*	*r*	*p*
MLD of the condyle (left)	−0.286	0.082	−0.390	0.016	−0.397	0.014	−0.380	0.019
MLD of the condyle (right)	−0.225	0.175	−0.172	0.303	−0.272	0.099	−0.126	0.453
MLD of the condyle (mean)	−0.270	0.535	−0.290	0.764	−0.352	0.329	−0.259	0.723
MLD of the condyle (left)	0.104	0.513	−0.050	0.848	0.163	0.704	0.059	0.768
MLD of the condyle (right)	0.109	0.101	0.032	0.077	0.064	0.030	0.050	0.117
MLD of the condyle (mean)	0.121	0.468	−0.009	0.956	0.127	0.446	0.062	0.712

As can be seen from Table 5, in the low angle group, FMA, FH‐OP, and SN‐OP were significantly correlated with the anteroposterior diameter of the condyle (right) and the anteroposterior diameter of the condyle, respectively, diameter (left) had a significant correlation and negative correlation; there was no significant correlation between FMA, SN‐OP, FH‐OP, and the MLD of the condyle.

**Table 5:** Correlation analysis within low angle group

Correlation	ANB		FMA		FH‐OP		SN‐OP	
	*r*	*p*	*r*	*p*	*r*	*p*	*r*	*p*
MLD of the condyle (left)	−0.540	0.134	−0.381	0.311	−0.590	0.095	−0.184	0.635
MLD of the condyle (right)	−0.536	0.137	−0.111	0.776	−0.444	0.231	−0.348	0.359
MLD of the condyle (mean)	−0.565	0.113	−0.256	0.505	−0.542	0.132	−0.281	0.464
MLD of the condyle (left)	0.053	0.893	−0.663	0.052	−0.429	0.250	−0.675	0.046
MLD of the condyle (right)	−0.295	0.442	−0.678	0.045	−0.719	0.029	−0.613	0.079
MLD of the condyle (mean)	−0.097	0.804	−0.702	0.035	−0.577	0.103	−0.68	0.043

*p < .05; **p < .01.

As can be seen from Table 6, in the angle equalization group, FH‐OP was negatively correlated with the MLD of the condyle; there was no significant correlation between FMA, SN‐OP, and FH‐OP and the anteroposterior diameter of the condyle.

**Table 6:** Intragroup correlation analysis of mean angle

Correlation	ANB		FMA		FH‐OP		SN‐OP	
	*r*	*P*	*r*	*P*	*r*	*P*	*r*	*P*
MLD of the condyle (left)	−0.375	0.125	−0.254	0.309	−0.328	0.184	−0.353	0.151
MLD of the condyle (right)	0.061	0.809	0.109	0.668	−0.668	0.002	−0.247	0.323
MLD of the condyle (mean)	−0.180	0.474	−0.081	0.749	−0.598	0.009	−0.355	0.148
MLD of the condyle (left)	0.191	0.448	0.360	0.142	−0.062	0.807	0.233	0.352
MLD of the condyle (right)	−0.091	0.720	−0.086	0.734	0.315	0.203	0.159	0.528
MLD of the condyle (mean)	0.072	0.777	0.184	0.465	0.139	0.582	0.241	0.334

*p < .05; **p < .01.

## 4 DISCUSSION

In the 1990s, CBCT was first used for oral and maxillofacial examinations in Japan and Italy. Because the cost of CBCT and radioactive agents is lower than CT, the scanning time is shorter, and the local image resolution is higher than that of CT. The advantages of high clinical diagnosis accuracy and flexible postprocessing software (Scarfe et al., 2006) provide a reliable and convenient method for the study of the skeletal structure of a TMJ and the clinical diagnosis and treatment of related diseases. Therefore, the CBCT observation method has been accepted by more and more dentists. This study also used CBCT to measure the anteroposterior diameter and the MLD of the condyle.

This study uses a functional occlusal plane. Of course, the steepness of the plane is also related to the relative excessive or insufficient eruption of the second molar in the vertical direction. At the same time, the wear of the tooth tip also has a certain error on the posthumous measurement results, which may affect the OP plane Accuracy of measurement data. However, the real occlusal plane is not a plane, but a curved surface. Therefore, from the perspective of this study, in the process of orthodontic treatment, facing patients with abnormal mandibular position, attention should be paid to the control of the vertical height of the posterior teeth and the consideration of the change of the inclination of the occlusal plane. The symmetry of the condyle includes symmetry in form and position. Symmetrical form refers to the symmetry of the condyle on both sides. The size is usually compared by measuring the anteroposterior diameter and the inner and outer diameters of the condyle. The type of deformity is significantly related. In this study, a separate study was conducted on skeletal Class II malocclusion patients, and the influence of differences in other malocclusion characteristics on the results of the study was excluded (Xiang et al., 2017).

Different types of malocclusion deformity due to the different occlusal relationships, the stress that the condyle bears are different, resulting in an adaptive alteration of the shape of the condyle (Katsavrias & Halazonetis, 2005). found that the mandibular rotation of patients with Class II and I classification was clockwise, mainly manifested as mandibular retraction, underdevelopment of the back height relative to the front height, and the short condyle. In patients with Class III, the mandibular rotation is counterclockwise to show a mandibular protrusion. The posterior height is overdeveloped relative to the front height, and the condyle is too long. Class I patients often have a straight face with an average angle, a high proportion of front and back is appropriate, and the development of condyle is normal. Park and other scholars believe that the abnormal scale makes the condyle of patients with Class II classification relatively flat, and the occlusal treatment can eliminate the morphological variation of the condyle and restore it to a normal circular contour (Park et al., 2014). Therefore, malocclusion of Class II and II classification should be treated as soon as possible, and the restriction of the upper jaw on the lower jaw should be lifted in time. Class II intermaxillary traction is used to guide the mandible forward so that the condyle moves forward to the normal middle position, and the position and shape of the joint will change accordingly, which can effectively prevent the occurrence of TMD. The research results of Haralur et al. (2014). confirmed that the anterior teeth of angle Class II malocclusion are prone to the risk of dendrite absorption due to the locked deep overbite, which leads to the overdevelopment of TMJ nodules and hinders the forward movement of the mandible, thus affecting the sagittal development of the mandible. Moreover, the condyle position is backward, the motion trajectory is irregular, and presents a typical twisted rope movement. Due to the abnormal occlusal relationship of skeletal Class II malocclusion, there is severe interference and mandibular movement disorder. The neuromuscular reflex guides the functional deviation of the mandible, which increases the burden of the TMJ and is very easy to cause TMD.

There was a significant difference between FMA and SN‐OP in skeletal Class II malocclusion, *p* < .05, and their sizes were high angle group > average angle group > low angle group. It is suggested that the size of FMA in skeletal Class II malocclusion is highly consistent with the development trend of SN‐OP. Growth and development are a concern for orthodontists. After Bjork (1969) proposed the concept of mandibular rotation, orthodontists began to realize the importance of mandibular rotation, the rotation of the lower jaw relative to the SN plane. At the same time, clinically, the author found that many patients with skeletal Class II malocclusion had mandibular rotation and the position of the condyle in the articular fossa had changed after plate treatment. It shows that the condyle has undergone adaptive reconstruction (i.e., the load stimulated the change of condyle shape). It also supports the results of this experiment that there is a significant correlation between the size of the condyle and occlusal plane, which is of great significance in orthodontic diagnosis and treatment.

From the mean value and correlation analysis of different angle groups, it can be seen that the MLD of the condyle is high angle group < mean angle group < low angle group. The analysis of the difference between the MLD of the condyle and the different angel groups is significant and negatively correlated, while the analysis of the difference between the anteroposterior diameter of the condyle and the different angel groups is not significant. Our conclusion is consistent with the scholar Kurusu et al. (2009), who proposed that in studying the relationship between masticatory force and the TMJ, he found that the long axis length of the condyle in the low‐angle group is greater than that in the high‐angle group, while the short axis length is in the different vertical skeletal face groups. There is no difference between them. Yao Shuang et al. (2012) measured the TMJ of type Class II adults with different vertical skeletal types, and found that the length of the long axis and the short axis of the condyle were both: low angle group > average angle group > high angle group. The results of this experiment are slightly different from those of Yao Shuang and other scholars.

The TMJ can be adaptively remodeled with changes in occlusion. Occlusion interference and mandibular dyskinesia caused by long‐term malocclusion can affect the shape and function of joints. Arnett and Gunson (2004) believes that the larger condyle is more stable when exercising the masticatory function, the matching degree of the condyle and the joint socket is higher, and this kind of condyle has a stronger ability to resist the occurrence of displacement. On the contrary, the small condyle does not provide enough support during occlusal changes, the connection with the joint socket is loose, and it is more likely to be displaced. The possibility of double or multiple occlusion is higher. The results of this study suggest: FMA, FH‐OP, and SN‐OP have a significant correlation with the MLDs of the condyle, and they are negatively correlated, that is, the larger angle of FMA, FH‐OP, and SN‐OP, the smaller the inner and outer diameter of the condyle. The diameter does not correlate with FMA, FH‐OP, and SN‐OP. From the measured mean values of the anteroposterior diameters and inner and outer diameters of the condyle, the high‐angle group was smaller than the average‐angle group and the low‐angle group. It suggests that patients with skeletal Class II high angle malocclusion may lack rotation fulcrum due to the steep occlusal plane, and the relative position relationship and occlusal characteristics of the mandible are special. Under the action of load, the condyle of the mandibular joint bear new stress stimulation, resulting in adaptive reconstruction in the shape and size of the condyle, which may be absorbed. That is, the mean value of condyle in patients with skeletal Class II high angle is the smallest, and finally has the typical characteristics of skeletal Class II high angle dislocation. At the same time, most patients with skeletal Class II malocclusion have mandibular hypoplasia. Whether the small sudden condyle is the cause of mandibular hypoplasia and whether there is a correlation between the two remains to be confirmed by further research.

Finally, in this study, it is difficult to collect case samples, and the average and low‐angle samples are relatively small, which may have a fixed impact on the accuracy of the experimental results. However, in terms of the starting point of this experiment and the data conclusions drawn so far, it has certain guiding significance for future clinical research and work. This study suggests whether orthodontists can promote the stability of the morphological structure of condyle by changing the inclination of the occlusal plane during the correction process, thereby maintaining a stable correction effect and thereby preventing and reducing the occurrence of TMD. Roth's functional occlusion theory believes that the goals of orthodontic treatment should include functional occlusion and TMJ health. We should pay close attention to the patient's occlusion and changes in the TMJ during the clinical correction. For the relationship between different malocclusion deformities and the shape and size of the condyle and the relationship between TMJ, clinical observations and further expansion of the sample should be combined to further deepen the research.

## 5 CONCLUSION

The FMA in skeletal Class II malocclusion is highly consistent with the development trend of the SN‐OP plane. The larger the FMA, FH‐OP, and SN‐OP among the groups, the smaller the MLDs of the condyle, which is not significantly related to the anteroposterior diameter of the condyle. In patients with skeletal Class II high angle malocclusion, the MLD and anteroposterior diameters of condyle were smaller than those of average angle and low angle groups, and negatively correlated with the FMA and SN‐OP. That is the steep occlusal plane, the smaller MLD of the condyle.

## AUTHOR CONTRIBUTIONS

Hainan Hu contributed to the design, search, and selection, and drafted the manuscript. Haisong Xu and Wanrong Lin contributed to the analysis and interpretation and critically revised the manuscript. Wu Jun and Hainan Hu contributed to the conception and design, search and selection, analysis and interpretation, and critically revised the manuscript. All authors gave final approval and agreed to be accountable for all aspects of the work ensuring integrity and accuracy.

## CONFLICT OF INTEREST

The authors declare no conflict of interest.

## Data Availability

The data sets generated and/or analyzed during the current study are not publicly available due to ethical requirements by the ethics committee.
